# Simple high‐resolution NMR spectroscopy as a tool in molecular biology

**DOI:** 10.1111/febs.14771

**Published:** 2019-02-14

**Authors:** Luca Mureddu, Geerten W. Vuister

**Affiliations:** ^1^ Department of Molecular and Cell Biology Leicester Institute of Structural and Chemical Biology University of Leicester UK

**Keywords:** ^15^N‐HSQC, CcpNmr AnalysisAssign, chemical shift mapping, NMR, Tstar

## Abstract

NMR is one of the major techniques for investigating the structure, dynamics and interactions between biomolecules. However, non‐experts often experience NMR experimentation and data analysis as intimidating. We discuss a simple yet powerful NMR technique, the so‐called chemical shift perturbation (CSP) analysis, as a tool to elucidate macromolecular interactions in small‐ and medium‐sized complexes, including protein‐protein, protein‐drug, and protein‐DNA/RNA interactions. We discuss current software packages for NMR data analysis and present a new interactive graphical tool implemented in CcpNmr AnalysisAssign version‐3, which can drastically reduce the time required for the CSP analysis. Lastly, we illustrate the usefulness of a protein three‐dimensional structure for interpretation of the CSP data.

**Abbreviations**
1D: one‐dimensional
2D: two‐dimensional
3D: three‐dimensional
BMRB: Biological Magnetic Resonance Data Bank
CcpNmr: collaborative computing project for NMR (software)
CSP: chemical shift perturbation
eq: equivalent
HSQC: heteronuclear single quantum coherence spectroscopy
NEF: NMR‐exchange format
ppm: parts per million
TROSY: transverse relaxation‐optimised spectroscopy
Tstar: testis‐signal transduction and activation of RNA

## Introduction

It is the ultimate aim of the molecular biologist to understand cellular functioning in its molecular context. As such, it is imperative to know at which time and place specific biomolecules are active to exert their function. At the root of our understanding, however, is the realisation that the interactions between individual molecules that together form active complexes of sometimes intricate complexity, constitute the underpinning basis of all the biological processes. Structural biology is the field of science which aims to describe such interactions between biologically relevant molecules at an atomic level. It is based on the notion that the interactions are facilitated by the specific molecular shapes and, as has nowadays become evident, also their dynamical changes. Together these are crucial in determining the affinities that drive the assembly of the macromolecular complexes 1.

**Figure 1:**
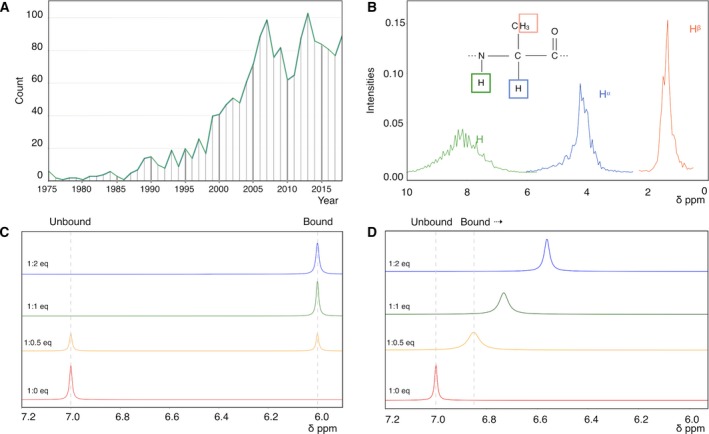
Chemical shift and exchange. (A) Number of CSP publications as function of year of publication. The plot shows the number of journal articles in the PubMed database by querying for ‘chemical shift (perturbation or mapping)’. (B) Distribution of deposited chemical shifts for the H^N^, H^⍺^ and H^β^ nuclei of Alanine as derived from the BMRB. For historical reasons, the scale in NMR is expressed in relative terms, the so‐called ppm scale, which runs from high positive values on the left to low, or negative values, on the right of the scale. (C) Simulated 1D ^1^ H^N^ NMR spectra under the slow chemical exchange regime. Spectra are shown at 0.0 (red), 0.5 (orange), 1.0 (green), and 2.0 (blue) eq of (NMR‐invisible) ligand. However, ∆δ was assumed to be −1 ppm (D) Simulated 1D ^1^ H^N^ NMR spectra under the fast chemical exchange regime. Spectra are coloured as previously. Peak positions were calculated using eqn (6) in Ref. 5, using ∆δ = −1 p.p.m., [protein] = 100 μm,* K*_d_ = 200 μm. Note the gradual shift of the peak as function of ligand concentration. The exchange‐induced broadening of peaks at 0.5 and 1 eq are slightly exaggerated for illustrative purposes.

NMR is one of the three major techniques that provides structural, dynamical and also interaction data 2. In this minireview, we will illustrate how a simple yet powerful experimental NMR technique, the so‐called chemical shift perturbation (CSP) analysis, can be used to investigate interactions between biomolecules or biomolecules and small drug‐like compounds. Since it was first proposed, the CSP analysis has become well‐established, as illustrated by the increasing number of papers referring to the technique (Fig. 1A), with currently ~ 80 references annually. In this paper, we also discuss how current NMR software packages can facilitate the CSP data analysis. Particular focus will be given to the CcpNmr AnalysisAssign version‐3, which provides several user‐friendly tools for retrieving the relevant data thus, providing for invaluable biological information.

## Chemical shift and exchange

NMR is a spectroscopic technique that employs an inherent property of many nuclei called ‘spin’ to yield spectra of various nuclei of biological interest, e.g. ^1^H, ^13^C, ^15^N and ^31^P. NMR's exquisite (bio‐)chemical usefulness originates from its ability to discriminate the different nuclei in a biomolecule. The electronic environment of each nucleus slightly modifies its exact resonance frequency through a process called chemical shielding and consequently the positions of various peaks in the NMR spectrum are specific for each nucleus in the molecule. The position of a peak in an NMR spectrum is commonly referred to as the ‘chemical shift’ and denoted by the symbol δ. The chemical shift constitutes one of the most important parameters and in NMR provides a powerful tool for a biochemist; as it not only allows us to discriminate one nucleus from another, but also provides information about their conformation and nearby chemical environment. For example, an analysis using recorded chemical shifts from the Biological Magnetic Resonance Data Bank (BMRB) reveals distributions with median values of ~ 8.24, ~ 4.32 and ~ 1.39 parts per million (ppm) for the Alanine H^N^, H^⍺^ and H^β^, respectively (Fig. 1B). The spread of each of the three distributions reflects the different conformations, i.e. the chemical environments, and dynamics, i.e. the change in these environments, of each of the nuclei in the various Alanine residues in their respective proteins.

In practice, the analysis of one‐dimensional biomolecular spectra is prohibitive because of spectral overlap. To overcome this problem, it is possible to correlate one nucleus with another, generating two‐dimensional (2D) or even higher‐dimensional [three‐dimensional (3D), 4D, nD] NMR spectra. For proteins in particular, a simple and very informative example is the 2D heteronuclear ^15^N‐heteronuclear single quantum coherence spectroscopy (HSQC) 3 (or alternatively for larger proteins the ^15^N‐TROSY‐HSQC 4) experiment. The resulting 2D ^15^N‐HSQC spectrum affords greater resolution and valuable information as peaks can be used as a ‘fingerprint’ of a protein. In practice, nearly each peak represents a backbone amide group of an individual residue, with the exception of peaks originating from the HN containing side chains of amino acids Asn, Gln, His, Trp, Lys and Arg. However, except for His, the signals from the side‐chain moieties are easily recognisable. Furthermore, proline residues are absent in 2D heteronuclear ^15^N‐HSQC spectra due to the lack of an amide group. The chemical shift values of the various nuclei can be extremely useful when used as a proxy to monitor protein–ligand interactions, a process called chemical shift mapping or CSP.

When a protein is titrated with a ligand, e.g. with a drug or another biomolecule, the chemical shift of the nitrogen and proton nuclei of the residues that are in close proximity to the binding site will be most affected. Thus, the binding of the ligand results in changes in the chemical shifts of these nuclei, causing the resulting peaks to alter their position in the NMR spectrum. By recording a series of NMR experiments at varying stoichiometries of protein and ligand, the resulting series of spectra conveys information regarding the affinity of the ligand, as well as identifying the important residues involved in the interaction.

To briefly explain the theoretical aspect of this phenomenon, consider for simplicity a peak for a nucleus *i* in a protein P at 7.0 δ ppm in a one‐dimensional (1D) NMR spectrum (Fig. 1C). For a simple two‐state binding process with ligand L:

(1) $$P+L\overset{k_{\text{on}}}{\underset{k_{\text{off}}}{⇌}}\text{PL}$$

the [P] : [L] = 1 : 0 equivalent (eq) condition (Fig. 1C, 1 : 0 eq) represents the left side of Eqn (1), where the protein is in its unbound state. Upon addition of a high‐affinity ligand, under the condition that the *k*_off_ is small, the equilibrium lies fully towards the bound state. Consequently, addition of the ligand causes the *i* peak in the unbound state to decrease in intensity, whereas a new peak at a different position appears; this new peak represents the bound state of the same protein nucleus *i*. At a [P] : [L] = 1 : 0.5 stochiometry (Fig. 1C, 1 : 0.5 eq) peaks for both the unbound and bound states will be present with equal intensities (neglecting dynamic effects). The difference between the bound‐ and unbound peak position is called ∆δ_*i*_. At a [P] : [L] = 1 : 1 stochiometry, only the peak of the bound state will be present, as the peak for the unbound state will have disappeared (Fig. 1C, 1 : 1 eq).

In NMR, the above situation when the *k*_off_ is much smaller than ∆δ_*i*_ is called the ‘slow exchange’ regime. In contrast, in a ‘fast exchange’ regime, when *k*_off_ is much greater than ∆δ_*i*_, in each of the spectra recorded at different [P] : [L] stochiometries, the peak position for nucleus *i* represents the population weighted average of its free and bound positions. Consequently, the peak appears to be ‘moving’ from its original position (Fig. 1D 1 : 0 eq) towards a its bound position as the ligand concentration increases (Fig. 1D, 1 : 0.5 eq to 1 : 2 eq). In cases where *k*_off_ ∼ ∆δ_*i*_, the peak typically disappears due to line broadening effects and this situation is called ‘intermediate‐exchange’ (not shown).

**Figure 2:**
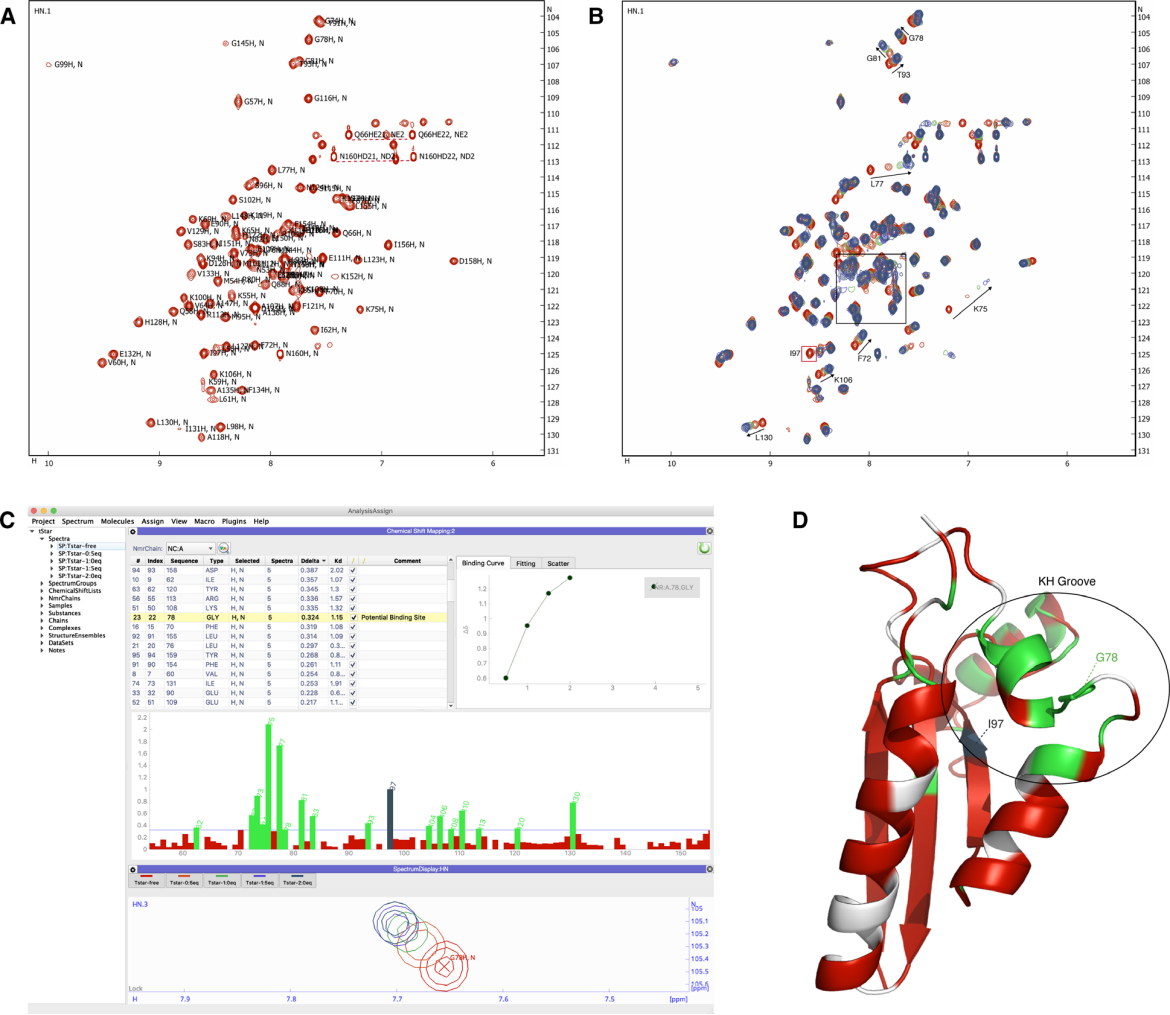
CSP analysis of the binding of Clip2 RNA (AAAUAA) to the Tstar‐KH domain 13. (A) ^15^N‐HSQC spectrum of 200 μm uniformly ^15^N‐labelled Tstar KH domain; selected assignments are indicated. (B) Five overlaid spectra of Tstar‐KH ^15^N‐HSQC domain. Spectra are shown at 0.0 (red), 0.5 (orange), 1.0 (green), 1.5 (purple) and 2.0 (dark blue) eq of Clip2 ligand. The black square box indicates the crowded region. Arrows indicate different peak perturbation trajectories. The I97 peak (red box) is present only at 0.0 eq but disappears upon addition of Clip2. (C) The CSP analysis module in AnalysisAssign version‐3. Clicking any bar in the bar chart (middle; included residues in green, excluded residues in red) or residue in the residue table (top) will navigate to the peaks of the corresponding residue in the spectra (bottom). The binding curve can be automatically displayed on the right side of the table. Multiple selection overlays related binding curves. All adjustments of parameters and settings of the CSP analysis module, such as setting the threshold line (i.e. horizontal line in middle panel) or excluding a residue from the analysis (checkboxes in the residue table), will result in a real‐time update of all plots without the need for any tedious or error‐prone manual actions. (D) Ribbon representation of the Tsar‐KH domain (PDB code 5EL3) with residues coloured according to their CSP values resulting from interaction of Clip2 RNA. Residues flagged with missing peaks in the spectra containing ligand, e.g. I97, are highlighted in dark blue. The black circle highlights the KH domain clip2 binding groove. Unassigned residues or residues removed from the analysis are indicated in light grey.

The real power of the CSP method lies in the identification of protein residues most affected by the ligand, i.e. those residues with nuclei that display large ∆δ_*i*_ values. In the case of multi‐dimensional spectra, such as the ^15^N‐HSQC spectrum (Fig. 2A), the total chemical shift change involving all dimensions is usually taken (*vide infra*, Eqn (2)) and used as a proxy for the importance of the specific residue in the interaction. Furthermore, for the fast exchange regime *K*_d_ can be determined from the observed ∆δ_*i*_ values as a function of ligand concentration 5.

## Chemical shift perturbation in practice

The first step in a CSP procedure is the assignment of individual peaks to a specific residue in the protein. Different software packages and algorithms have been developed to establish the assignments of the backbone nuclei, i.e. H^N^, N, C^α^, C’, either by manual or automated approaches 6. According to Lee and Markley, based on BMRB statistics in 2014, Sparky was still the most widely used NMR data analysis tool for backbone assignment 7. A much more recent programme, not included in their list, is CcpNmr AnalysisAssign version‐3 8, which provides tools for simple and semi‐automated backbone assignments and a dedicated interactive CSP analysis module, from here on referred to as the CSP module (Fig. 2C). An *ab initio* protein backbone assignment can be a time‐limiting factor, as it requires some effort in terms of sample preparation, NMR spectra recording and data analysis. However, assignments can potentially be retrieved from databases, such as the BMRB, and serve as the starting point, adjusting them as required by the user's experimental conditions.

Table 1 displays an overview of functionalities from the various NMR data analysis programmes that are relevant to a CSP analysis. After the initial setup, either Sparky 7, CcpNmr Analysis version‐2 9, NMRView 10 and cara 11 can automatically calculate the ∆δ from chemical shifts. However, only CcpNmr AnalysisAssign V3 has capabilities for interactive inspection of the ∆δ data in relation to the underpinning spectra, interactive thresholding and easy adaptation of various relevant parameters. Moreover, apart from AnalysisAssign version‐3 users will have to manually plot and/or export to other software packages for further analysis. This typically will involve multiple manual steps before retrieving the biological relevance from their initial NMR dataset. In addition, parameter adjustments will necessitate repeating these various steps, thus increasing the time required for the whole analysis and inevitably increasing the risk of introducing human errors. The AnalysisAssign version‐3 CSP module, contains all required functionalities for a full CSP analysis, including automatically generated bar charts of CSP values as function of residue number which are linked to the underpinning spectra, peak tables and a binding plot. Additionally, if the protein molecular structure information is available, the annotations and selections can be mirrored to a graphical molecular visualisation, such as pymol 12.

**Table 1:** Comparison of common freely available NMR software packages with built‐in backbone assignment (as shown on the BMRB statistics in 2014, 7) and CSP analysis capabilities

Features	CcpNmr V3	CcpNmr V2	Sparky	NMRView/CARA
Peak automation^a^	Simultaneous peak selections, copying and re‐fitting	Simultaneous peak selections, copying and re‐fitting	Simultaneous peak selections, copying and re‐fitting	Single peak re‐fitting
Interactivity^b^	Selectable plot items and tables with live updates	None or static plots	None	None
Settings^c^	Multiple dimensionality	Limited dimensionality	Limited dimensionality	Limited dimensionality
	Multiple atoms			
	Multiple ∆δ calculation modes			
	Several GUI parameters			
Extras^d^	Link to molecular visualisation	None	User's macros	User's macros
	IPython console			
	Macro editor			
Exports^e^	Images: various formats	Text: various formats	Text: various formats	Text: various formats
	Texts: various formats			
	Software readible: Json			

a Graphical peak selection, copying assignments between spectra, peak adjustment and refitting, provisions to follow peaks across titration series.

b Live updates of results, interactive adjustment of parameters.

c Adaptable to different experiment types, ability to handle different dimensionalities, ability to handle different peak parameters.

d Interaction graphical visualisation tools, ability to link to other software packages.

e Exports to external formats.

We tested the AnalysisAssign CSP module by exploring the binding of Clip2 RNA, AAAUAA, to the testis‐signal transduction and activation of RNA (Tstar)‐KH domain 13, 14. We imported both the assignments of the free Tstar‐KH domain in addition to a series of five ^15^N‐HSQC spectra of uniformly ^15^N‐labelled Tstar‐KH at 0.0, 0.5, 1.0, 1.5, and 2.0 eq of Clip2 ligand, directly from the original Sparky data using the inherent data conversion routines of AnalysisAssign. The assignments of the KH domain were propagated from the spectrum at 0.0 eq of ligand to all other spectra using the simple drag and drop feature of AnalysisAssign for copying peak lists. As expected, some of the peaks had changed their positions upon titration with the ligand. Using the interactive tables and spectrum displays it was possible to easily identify shifted peaks and correct their position in the target spectra, either individually, or as a group of peaks across multiple spectra, or automatically. Figure 2B shows the overlay of the five spectra, with trajectories of shifted peaks indicated for selected residues.

Figure 2C shows the overlay of the five ^15^N‐HSQC spectra at the position of G78. The gradual change in peak position upon increasing ligand concentration is evident. Importantly, to ensure a valid analysis all spectra should be properly referenced as changes in peak positions could otherwise be misinterpreted. Fortunately, AnalysisAssign has routines to establish, and where needed report, on the spectral alignment that functions even in the case of non‐fully identical spectra 8. Using an automated analysis, in which all the peaks were accurately matched to their extrema, CSP_*i*_ values are calculated for each Tstar‐KH residue *i* using Eqn (2) and displayed automatically as a bar plot in the CSP module interface.

(2) $${\text{CSP}}_{i}=\sqrt{{\left(\Delta \delta_{\mathrm{Hi}}\right)}^{2}+\alpha {\left(\Delta \delta_{\mathrm{Ni}}\right)}^{2}}$$

where α denotes the relative weighting of chemical shift changes of the ^15^N nuclei relative to the ^1^H nuclei, by convention set to 0.14 15, and ∆δ_*Hi*_ and ∆δ_*Ni*_ denote the observed changes of the proton and nitrogen chemical shifts for residue *i*, respectively. Crucially, the threshold below which the CSP_*i*_ values are deemed not significant needs to be established. A value of 1σ derived from the distribution of all CSP values is set by default as the first estimate without a need of other filters 5. Rapid manual inspection of the affected residues establishes if this threshold needs upward or downward adjustment. In the case of G78, the peak follows a consistent trajectory upon ligand titration with well‐defined changes in peak positions, rendering its CSP an appropriate threshold value. In contrast, another residue with a similar CSP value, A138, is located in a crowded region of the spectrum (cf. Fig. 2B) and thus its peak movements in such areas are potentially compromised by mis‐assignment of the peaks. Such residues should be flagged and we recommend that, barring further NMR data confirming their proper assignment, they first be excluded from the analysis. In our view it is wise to reduce any possible false positives, until the moment that their inclusion appears warranted, e.g. after careful inspection of the structure (*vide infra*) or on the basis of other data that confirms that they can be considered relevant for the binding event. Depending on *k*_off_ and the residue‐specific ∆δ_*i*_, peaks can disappear in any of the spectra with ligand concentrations > 0. Such situation still conveys useful information about the involvement of the specific residue, albeit that the exact magnitude of its CSP_*i*_ value cannot be established. In general, the disappearance of a peak for a specific residue is assumed to imply a ∆δ_*i*_ (CSP_*i*_) value resulting in exchange broadening due to an intermediate exchange regime and hence signifies importance. Consequently, these residues are automatically flagged by the CSP analysis module, e.g. residue I97 in Tstar‐KH.

The true power of the CSP analysis is revealed when mapping the CSP results onto a molecular structure. The CSP module can automatically map the annotations and residue selections onto a molecular structure of the biomolecule under investigation using an external molecular visualisation programme, such as pymol 12. The CSP analysis of the interaction of Clip2 RNA with the Tstar‐KH domain identified residues F72, V73, G74, K75, L77, G78, G81, S83 T93, I97, R104, K106, K108, E110, R113, Y120 and L130 with significant CSP values (Fig. 2C). When mapped onto the structure of Tstar‐KH, a clustering of several affected residues is observed across an interface formed by one α‐helix, two ß‐strands and two loops, corresponding to the KH hydrophobic groove (Fig. 2D). In fact, according to Feracci *et al*. 13 residues G78 and I97, (the latter flagged as ‘missing’ by the CSP analysis), belong to a set of crucial residues in the groove that stabilise the interaction with the Clip2 RNA.

In cases where no 3D structure is available, often the structures of homologous proteins can be used as a reference. Protein structure is more highly conserved than primary sequence; therefore, small changes in protein composition usually do not significantly alter the structure of the macromolecule. Alternatively, in many cases, protein structures can be fairly accurately obtained using homology modelling, where an existing structure of a homologous protein is used as a template to generate the structure of the protein of interest. Such models can be reliably used for mapping of protein–ligand interactions, assuming the binding interface has not been affected by the mutations. A description of contemporary available homology modelling software packages and servers are discussed in the review by Vyas *et al*. 16.

Currently freely available NMR software suites, such as Sparky, allow retrieving CSP values from their tables, but users are required to manually export to third party software to carry on the analysis or are limited to single and static plotting, like in the case of CcpNmr Analysis version‐2. The possibility to graphically and interactively inspect the NMR data that identify the residues involved in protein–ligand interactions, makes the new AnalysisAssign CSP module extremely useful for non‐experts and drastically reduces the time required for a CSP analysis. The CSP module is also not limited to ^1^H‐^15^N as it can accommodate any combination of nuclei, e.g. ^1^H–^13^C in case of methyl residues. AnalysisAssign is implemented in a flexible fashion that will facilitate easy adaptation to insert specific calculation modes for ∆δ_*i*_ values, automatic pre‐ and user‐defined *K*_d_ fittings as well as direct links to external auto‐docking software such as HADDOCK 17. For more advanced users, it is also possible to use the AnalysisAssign libraries to create specific, but simple macros to extrapolate further information from the dataset (Table 1). For example, a macro can be used to plot the minimal shift changes resulting from the mutation of a specific residue in a protein, including in this calculation only residues in which the CSP is above the defined threshold value. Several settings and data exporters based on the NMR‐exchange format (NEF, 18) or tabular.xls format have been implemented, thus providing tools to easily export information to other programs for further analysis if so required.

A series of other programmes have also been developed to address specific tasks using peaks in NMR spectra and NMR assignments, such as the programme Farseer, which performs analyses on large and multivariable datasets, including CSP 19. Other programmes include auto‐FACE, which facilitates the identification of binding mechanisms from CSP data 20 and TITAN, which uses peak perturbations trajectories to help the identification of interaction mechanisms 21.

## Conclusions

Chemical shift perturbation is very useful as a simple tool to elucidate macromolecular interactions in small‐ and medium‐sized complexes, including protein–protein, protein–drugs, and protein–DNA/RNA interactions 5, either in solution, or in solid state NMR 22. The CSP method works best when recording heteronuclear NMR spectra on samples in which the biomolecule, e.g. the protein, is isotopically labelled as to allow for selective detection. Fortunately, protein overexpression and isotope labelling, e.g. by ^15^N or ^13^C, has now become routine in *Escherichia coli* and *Pichia Pastoris*, with new and promising developments for expression in higher eukaryotic systems, which guarantees the presence of a more complex folding machinery and post‐translational modifications 23. Together with new developments in NMR technology, e.g. direct ^15^N‐observation 24, the CSP method will find even more widespread application.

In comparing the various programmes for CSP data analysis, we find that the CSP module of AnalysisAssign version‐3 provides for a simple and interactive graphical tool that allows users to significantly reduce the time required for a CSP analysis.

## Downloads

AnalysisAssign can be freely downloaded for non‐commercial academic usage from http://www.ccpn.ac.uk/v3-software/downloads. The CSP module and tutorials will be included in its upcoming version 3.0 release.

## Conflict of interest

The authors declare no conflict of interest.

## Author contributions

LM designed and developed the CSP analysis module for CcpNmr AnalysisAssign Version 3.0. LM & GWV analysed the data. LM & GWV wrote the manuscript.
